# Enhanced event-based surveillance for imported diseases during the Tokyo 2020 Olympic and Paralympic Games

**DOI:** 10.5365/wpsar.2021.12.4.903

**Published:** 2021-12-22

**Authors:** Ayu Kasamatsu, Masayuki Ota, Tomoe Shimada, Munehisa Fukusumi, Takuya Yamagishi, Anita Samuel, Manami Nakashita, Tomohiko Ukai, Katsuki Kurosawa, Miho Urakawa, Kensuke Takahashi, Keiko Tsukada, Akane Futami, Hideya Inoue, Shun Omori, Miho Kobayashi, Hiroko Komiya, Takahisa Shimada, Sakiko Tabata, Yuichiro Yahata, Hajime Kamiya, Fumi Yoshimatsu, Tomimasa Sunagawa, Tomoya Saito

**Affiliations:** a Field Epidemiology Training Program, National Institute of Infectious Diseases, Tokyo, Japan.; b Center for Field Epidemic Intelligence, Research and Professional Development, National Institute of Infectious Diseases, Tokyo, Japan.; c Center for Emergency Preparedness and Response, National Institute of Infectious Diseases, Tokyo, Japan.

## Abstract

In 2021, the National Institute of Infectious Diseases, Japan, undertook enhanced event-based surveillance (EBS) for infectious diseases occurring overseas that have potential for importation (excluding coronavirus disease 2019 [COVID-19]) for the Tokyo 2020 Olympic and Paralympic Summer Games (the Games). The pre-existing EBS system was enhanced using the World Health Organization Epidemic Intelligence from Open Sources system and the BlueDot Epidemic Intelligence platform. The enhanced EBS before and during the Games did not detect any major public health event that would warrant action for the Games. However, information from multiple sources helped us identify events, characterize risk and improve confidence in risk assessment. The collaboration also reduced the surveillance workload of the host country, while ensuring the quality of surveillance, even during the COVID-19 pandemic.

Due to the coronavirus disease 2019 (COVID-19) pandemic, the Tokyo 2020 Olympic and Paralympic Games (the Games) were rescheduled for 23 July to 5 September 2021. The attendance of spectators from abroad was not permitted; however, several tens of thousands of people associated with the Games were expected to visit Japan from more than 200 countries and regions. The visitors included national Olympic and Paralympic team members, media crews and sponsors. Since international mass gatherings have high potential to disseminate communicable diseases to several countries, (*1*) it was important during the Games to monitor infectious diseases occurring overseas that have potential for importation.

Event-based surveillance (EBS) is the organized and rapid capture of information about events that are a potential risk to public health. (*2*) Official and unofficial information sources can be used for EBS, and the information obtained should be used to rapidly assess the risk that the event poses to public health, so that a timely response can be taken. As stated in the Asia Pacific Strategy for Emerging Diseases and Public Health Emergencies (APSED III), (*3*) various information sources for EBS are useful for assessing contextual vulnerabilities and creating risk assessments to develop response strategies.

In the past, new EBS systems have often been created for international mass gatherings to respond to complex and evolving situations. However, this was not practical for the Games, owing to the burden of the COVID-19 pandemic on national surveillance and response teams. Therefore, to address the high demand on local resources, we used external resources in our enhanced EBS for imported infectious diseases. This paper describes the methodology and preliminary results of the enhanced EBS for infectious diseases occurring overseas (excluding COVID-19) that have potential for importation before and during the Games.

## Methods

The enhanced EBS for the Games was conducted at the National Institute of Infectious Diseases (NIID), Japan, which houses the country’s Field Epidemiology Training Programme (FETP). Three staff members and 15 FETP fellows were engaged in EBS from 1 July to 19 September 2021, 7 days a week. The initial period (1–10 July) was a test run, during which EBS was conducted in the same way as for the actual operation (from 11 July). Each day, two fellows and one staff member oversaw the daily EBS. Concurrent national disease surveillance systems, including those for COVID-19 and EBS for domestic events, are not described here.

**Figure 1:**
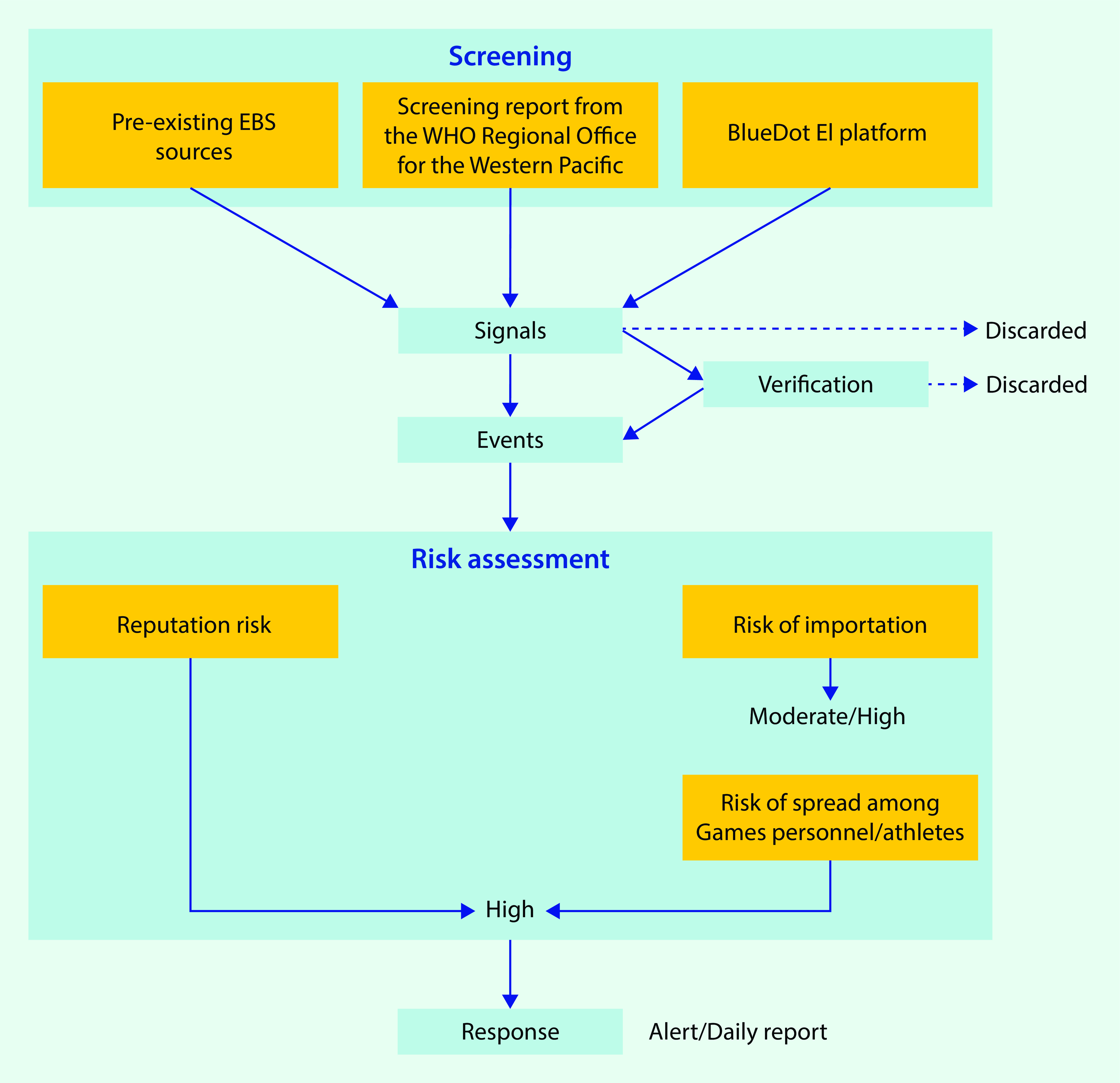
Overview of event-based surveillance for infectious diseases occurring overseas^a^ during the Tokyo 2020 Olympic and Paralympic Games, Japan

The enhanced EBS systems supplemented an existing surveillance system targeting 69 diseases in 80 countries, not including Japan (**Table 1**). The priority diseases were pre-selected based on their epidemic status, severity and unfamiliarity among physicians in Japan (a factor that may cause delays in diagnosis and treatment). The countries and regions to be monitored were selected from among those that have previously participated in the Games with the highest numbers of estimated participants and officials present. The enhanced EBS for infectious diseases occurring overseas (other than COVID-19) comprised the pre-existing EBS system plus two external systems – the World Health Organization (WHO) Epidemic Intelligence from Open Sources (EIOS) system and the BlueDot Epidemic Intelligence (EI) platform, a surveillance and risk assessment platform that leverages both artificial intelligence and human intelligence (**Fig. 1**). (*4*, *5*)

[**insert** Figure 1]

**Table 1:** List of priority infectious diseases (other than coronavirus disease 2019) for event-based surveillance during the Tokyo 2020 Olympic and Paralympic Games, Japan (*n* = 80)

Mode of transmission	Surveillance-priority infectious diseases
Human-to-human	Diphtheria, poliomyelitis, tuberculosis,^a^ hepatitis B,^a^ varicella, pertussis, measles, rubella, sexually transmitted infections (HIV, syphilis, chlamydia, gonorrhoea),^a^ meningococcal disease, seasonal influenza, acute gastroenteritis, mumps, bacterial meningitisa
Foodborne	Enterohaemorrhagic *Escherichia coli* infection,^a^ cholera, shigellosis,^a^ typhoid/ paratyphoid, hepatitis A, hepatitis E, botulism, amoebiasis,^a^ cryptosporidiosis,^a^ giardiasis,^a^ listeriosis
Soil/waterborne	Coccidioidomycosis, leptospirosis, Legionnaires’ disease, melioidosis, tetanus, Cryptococcus gattii infection,^a^ strongyloidiasis, histoplasmosis
Animal-borne	Middle East respiratory syndrome coronavirus, lassa fever, South American haemorrhagic fever, avian influenza, Q fever, rabies, anthrax, hantavirus infection, brucellosis, hendra virus disease, Rift Valley fever, tularaemia, lyssavirus infection^a^
Mosquito-borne	Japanese encephalitis, West Nile virus infection, yellow fever, Zika virus disease, chikungunya virus disease, Western equine encephalitis, Eastern equine encephalitis, dengue, malaria, St. Louis encephalitis, La Crosse encephalitis, Ross River virus disease, Barmah Forest virus disease, Oropouche fever
Tick-borne	Severe fever with thrombocytopenia syndrome virus infection, Crimean-Congo haemorrhagic fever, tick-borne encephalitis, Lyme disease, Omsk haemorrhagic fever, recurrent fever, Kyasanur Forest disease, Colorado tick fever, Rocky Mountain spotted fever, African tick-bite fever,^a^ Queensland tick typhus,^a^ Mediterranean spotted fever, other spotted fever group rickettsioses,^a^ Powassan virus disease, anaplasmosis, ehrlichiosis
Other arthropod-borne	Plague, scrub typhus, leishmaniasis, Chagas disease

^a^ Diseases not included in the BlueDot system.

Pre-existing EBS sources included International Health Regulations (2005) notifications and information publicly available via the Internet. Sources included official information from international organizations such as WHO and national health authorities, and unofficial information from news aggregators, blogs, expert groups and other systems such as ProMED, the Center for Infectious Disease Research and Policy at the University of Minnesota and HealthMap. From these sources, we screened for events each day based on our screening criteria (**Box 1**).

Box 1 **Screening criteria for pre-existing event-based surveillance, Japan, 2021**

Events related to emerging infectious diseases that should be monitored:

sustained human-to-human transmission of a known emerging infectious disease
outbreaks with undiagnosed symptoms.

Events of concern that have potential impact on  Japan:

events with potential impact on Japanese travellers
events with potential for disease importation (occurrence above baseline, unexpected outbreaks of fatal infectious diseases)
events with contaminated food distributed to Japan
potential for dispatch of international emergency relief teams from Japan
potential need to review response and countermeasures (e.g. update of case definitions, update of epidemiological investigation guidelines).
Events posted on WHO event information site

The WHO EIOS system is a web-based system designed to augment and accelerate global public health intelligence activities. (*6*) It collects articles each day from a broad range of online official and unofficial sources and publishes the categorized information through its user interface, which is accessible only to authorized individuals. The WHO Regional Office for the Western Pacific conducted screening based on their standardized approach and e-mailed NIID a list of detected signals once a day. (*4*) This screening report provided a summary of signals, including the number of reports, affected population characteristics, reporting period, reporting region, baseline data and actions taken. The Regional Office also provided their qualitative assessment of the risk of importation into Japan during the Games and of further spread within the country, as well as the potential significant impact on society. These signals were defined as events.

BlueDot’s web-based EI platform shows quantitative risk assessments based on modelling that calculates the importation risk based on air travel data and local infectious disease epidemiological data. (*7*, *8*) BlueDot obtained local disease activity hourly from online sources such as international organizations and public health agencies, ProMED-mail and Global Database of Events, Language and Tone. The information was first scanned by BlueDot’s artificial intelligence system and then screened and verified by their experts. Users could select a disease on the platform and see the risk of importation from every other country to Japan. The risk of importation was defined by BlueDot as at least one infected person entering Japan by plane and was classified as high or higher risk if it was greater than 50%. We checked the platform for updates at a set time each day and defined events as those that were newly flagged as high or higher risk.

For signals or events with uncertain information, verification was conducted by referring to official sources or by combining multiple sources. After verification, we recorded all events in the EBS database and conducted risk assessments to determine their risk of association with the Games (**Box 2**). First, we assessed the potential risk of importation of diseases to Japan in relation to the Games by referring to previous national surveillance data of imported cases to Japan, (*9*-*11*) WHO epidemiological reports, the number of previous visitors to Japan (*12*) and the number of estimated Games participants. Second, if an importation risk related to the Games existed, the consequent risk of transmission among Games personnel and athletes was evaluated. We also assessed whether events posed a potential risk to the Games. The level of risk was discussed between staff and FETP fellows and was qualitatively determined as high, medium or low by consensus.

Box 2 **Risk assessment criteria for publishing events in the daily National Institute of Infectious Diseases report, Japan, 2021**

Does the event have a high probability of importation of infectious diseases?

Do the infectious diseases have a high probability of transmission among Games personnel?
Do the infectious diseases have a high probability of transmission from Games personnel to the community?

Does the event have a reputational risk among Games personnel and relevant stakeholders?

Through these processes, events that were considered to pose a high risk to the Games were posted in daily reports with summaries and assessments. They were distributed to local governments and the Tokyo Organizing Committee of the Olympic and Paralympic Games through the Ministry of Health, Labour and Welfare, to alert them and help them to respond in a timely manner.

## Results

Overall, 140 events and 20 diseases were identified by the enhanced EBS system during the provisional period of 11 July to 8 August 2021; that is, from the end of the 10-day test run to the closing day of the Olympics (**Table 2**). A total of 17 events and 10 diseases were detected by the pre-existing system, 121 events and 11 diseases by the EIOS system, and two events and two diseases by the BlueDot platform. The median number of events per day was 5 (range, 1–9).

**Table 2:** Number of events and diseases detected by event-based surveillance of infectious diseases occurring overseas^a^ before and during the Tokyo 2020 Olympic Games, Japan, 11 July to 8 August 2021

	Pre-existing EBS	Screening report from the WHO Regional Office for the Western Pacific	BlueDot EI platform	Total
Number of events	17	121	2	140
Number of diseases	10	11	2	20
Disease	Avian influenza B virus infection, Cyclospora infection, cholera, dengue, Japanese encephalitis, Middle East respiratory syndrome, monkeypox, plague, typhoid fever	Acute gastroenteritis, chikungunya, dengue, hepatitis A, hepatitis B, Middle East respiratory syndrome, sexually transmitted infections, unknown disease, West Nile virus infection, yellow fever, Zika virus disease	Dengue, malaria	

EBS: event-based surveillance; EI: epidemic intelligence; WHO: World Health Organization.

^a^ Excludes coronavirus disease 2019.

All identified events were evaluated for risk, with none meeting the high-risk criteria for publishing in the daily report (**Table 3**). The time required to conduct EBS using the three systems was less than 60 minutes per FETP fellow per day.

**Table 3:** Examples of risk assessment for events detected in event-based surveillance before and during the Tokyo 2020 Olympic Games, Japan, 11 July to 8 August 2021

Date of recording	EBS system/disease/source	Event summary	Risk assessment
29 July	EIOS/hepatitis A/media	495 cases associated with a national hepatitis A outbreak have been reported in North Carolina, USA, since 1 January 2021.	The USA has been experiencing nationwide outbreaks of hepatitis A since 2017, spread through person-to-person contact. The number of imported cases detected in Japan from the USA over recent years has been 0–2 per year. The number of people entering Japan from the USA has significantly decreased, and the risk of travellers, including Games personnel, importing the virus into Japan is low.
29 July	Pre-existing EBS/monkeypox/WHO Disease Outbreak News	A patient who developed monkeypox travelled from the USA to Nigeria on 25 June. He returned to the USA on 9 July after disease onset and was quarantined on 13 July. Possible community and health-care contacts are being monitored. The source of infection for this case is unknown.	The risk of importation from Nigeria to Japan is low due to a significant decrease in the number of travellers and the low number of Games participants from Nigeria. The risk of spread of infection in the USA is low because contacts in the USA had been identified and were monitored during the incubation period after their last contact date. Therefore, the risk of importation into Japan is low.
3 August	BlueDot EI platform/malaria/media	377 599 new cases of malaria were recorded in the northern Angolan province of Malanje in the first half of 2021, resulting in the deaths of 268 people. This is an increase in cases, but a reduction in deaths, compared with the same period in 2020.	The actual increase in cases cannot be determined because data for previous years were not available. There have been no imported malaria cases from Angola in the past 5 years, the number of travellers has decreased significantly from recent years, and the number of Games participants from Angola is less than 50. Therefore, the risk of importation into Japan is low.

EBS: event-based surveillance; EI: epidemic intelligence; USA: United States of America; WHO: World Health Organization.

## Discussion

Enhanced EBS of infectious diseases occurring overseas that have potential for importation, other than COVID-19, was conducted for the Tokyo 2020 Olympic and Paralympic Games using the pre-existing EBS system and external EBS systems. The provisional results revealed that no events occurring overseas were assessed as high risk for importation during the Games and none qualified to be published in the daily report. The absence of such events during the Games may be due to reports of imported infectious diseases decreasing during the pandemic. (*13*) Although travellers entered Japan for the Games, overall arrivals were substantially lower than before the COVID-19 pandemic, which may have led to an overall decrease in importation risk. In addition, infection control measures in place against COVID-19 may have decreased the risk of disease importation.

The enhanced EBS for the Games resulted in more reliable risk assessments because the framework incorporated data triangulation among three sources – the pre-existing EBS system in Japan, the WHO EIOS system and the BlueDot web-based EI platform. The same signals, obtained from multiple articles and different sources, were often reported from each system; such consistency in signals coming from sources with different timeliness, representativeness, sensitivity and completeness may increase the validity of risk assessments. Furthermore, the intelligence obtained from different sources was complementary, providing more detailed information about the event than relying on a single source, which may have contributed to appropriate risk assessment. (*3*)

Using three EBS systems also prevented public health events from being missed. For example, signals obtained from one system were not picked up as events in the other systems. This was partly due to differences in the initial assessment (e.g. BlueDot could conduct quantitative risk assessment using more accurate travel data, whereas the EIOS-based screening report qualitatively assessed the risk associated with the Games).

Incorporating external surveillance systems had the potential to reduce the time and effort required for signal screening for the Games. The EIOS system is a useful tool to deliver extensive and prompt information, but its informative nature makes it time consuming. Previously, the EIOS system was used for the 2019 Rugby World Cup in Japan, with 79 infectious diseases across 30 countries targeted for surveillance; it required one staff member and two FETP fellows to work for 3 hours each day. (*14*) For the Games, a larger number of countries were targeted; however, the time required was less than  1 hour per day per FETP fellow. This reduction in time was largely due to events being triaged by the Regional Office and BlueDot, which allowed NIID staff to rapidly initiate an assessment based on information provided. Globally, public health resources have been limited during the COVID-19 pandemic; hence, the technical support from external resources was vital for implementing enhanced surveillance for the Games. During future mass gatherings, the use of external platforms may make EBS more efficient for local governments and facilities with limited human resources.

There were limitations to this enhanced EBS in terms of data triangulation. First, many of the information sources used by the three systems overlapped because they obtained information through existing informal or formal channels such as social media or ProMED. Second, since the newly adopted systems were outsourced, there was a time lag between the signal screening and our detection. These limitations need to be considered if the assessment and response are required immediately, in which case, the system would need to be based at the relevant internal institution.

EBS to monitor infectious diseases occurring overseas, apart from COVID-19, for the Games in Japan was enhanced by working with external organizations. The triangulation of information provided reliable risk assessments without missing significant events. Furthermore, the collaboration helped to reduce the effort required to screen a wide range of sources internally while maintaining the quality of surveillance, especially for this event that occurred during the COVID-19 pandemic.
